# Inhibition of Wnt signaling and cancer stem cells

**DOI:** 10.18632/oncotarget.309

**Published:** 2011-08-21

**Authors:** Desheng Lu, Dennis A. Carson

**Affiliations:** University of California San Diego (UCSD) Moores Cancer Center, La Jolla, CA 92093-0820, USA

Cancer stem cells represent a small number of pluripotent and self-renewing cells within a tumor, which are resistant to conventional chemotherapy and are responsible for tumor initiation and maintenance. The Wnt/β-catenin signaling pathway drives stem cell self-renewal and is involved in the pathogenesis of various types of cancer. Aberrant activation of the Wnt signaling pathway in normal stem cells can promote their transformation into cancer stem cells [1,2]. Thus, drugs targeted to the Wnt/β-catenin pathway may potentially be effective in eliminating cancer stem cells. Recently, several small molecule Wnt signaling inhibitors have been identified, that can have therapeutic potential against cancers associated with aberrant Wnt signaling [3]. However, most reported Wnt inhibitors are still in the developmental stage, due to lack of specificity and a poor understanding of their precise mechanisms of action. Furthermore, there is limited evidence that these Wnt signaling inhibitors can eradicate cancer stem cells, without depleting normal stem cells in the bone marrow and the gastrointestinal tract.

Salinomycin, an antibiotic potassium ionophore, is the first drug reported to act as a selective breast cancer stem cell inhibitor. It kills breast cancer stem cells at least 100 times more effectively than paclitaxel in mice, but is relatively non-toxic to normal stem cells [4]. The mechanism of salinomycin action in the cancer stem cells remains unclear. Our recent studies have shown that salinomycin potently inhibits proximal Wnt/β-catenin signaling. It blocks the phosphorylation of the Wnt co-receptor LRP6, and induces its degradation [5]. Considering the importance of Wnt signaling in stem cell biology, the Wnt antagonistic action of salinomycin may contribute to its selective toxicity toward breast cancer stem cells. It will be interesting to test whether other Wnt signaling inhibitors with targets downstream of LRP6 are able to kill selectively breast cancer stem cells.

A successful anticancer therapeutic regime should eliminate both the differentiated cancer cells and the cancer stem cell population. Classical cytotoxic agents may deplete the bulk of a cancer but not the inherently chemo-resistant cancer stem cells, which ultimately recur and metastasize. The molecularly targeted agents act on aberrant molecular pathways associated with tumor development and progression, and have been successfully introduced to treat patients with various cancers [6]. These drugs include Bcr-abl inhibitors, epidermal growth factor receptor (EGFR) inhibitors, vascular endothelial growth factor (VEGF) antagonists, and recently Raf kinase and Alk kinase inhibitors. These single-target agents have spectacularly initial clinical efficacy when used alone, but none are able to cure a cancer. Future experiments need to determine if the combination of a single-target agent with salinomycin or other Wnt signaling inhibitors might have the ability to induce curative response in some tumors.
